# A Fit-Fat Index for Predicting Incident Diabetes in Apparently Healthy Men: A Prospective Cohort Study

**DOI:** 10.1371/journal.pone.0157703

**Published:** 2016-06-24

**Authors:** Robert A Sloan, Benjamin A Haaland, Susumu S Sawada, I-Min Lee, Xuemei Sui, Duck-chul Lee, Yassine Ridouane, Falk Müller-Riemenschneider, Steven N Blair

**Affiliations:** 1 Kagoshima University, Graduate Medical and Dental School, Department of Psychosomatic Internal Medicine, Kagoshima, Japan; 2 Georgia Institute of Technology, H. Milton Stewart School of Industrial and Systems Engineering, Atlanta, Georgia, United States of America; 3 National Institute of Biomedical Innovation, Health and Nutrition, Department of Health Promotion and Exercise, Tokyo, Japan; 4 Harvard University, Harvard T.H. Chan School of Public Health, Department of Epidemiology, Division of Preventive Medicine, Department of Medicine, and Brigham and Women’s Hospital and Harvard Medical School, Boston and Cambridge, Massachusetts, United States of America; 5 University of South Carolina, Arnold School of Public Health, Department of Exercise Science, Columbia, South Carolina, United States of America; 6 Iowa State University, College of Human Sciences, Department of Kinesiology, Ames, Iowa, United States of America; 7 National University of Singapore, Saw Swee Hock School of Public Health, Singapore; Iran University of Medical Sciences, ISLAMIC REPUBLIC OF IRAN

## Abstract

**Background:**

The purpose of this study was to examine the impact of combined cardiorespiratory fitness and waist-to-height ratio in the form of a fit-fat index on incident diabetes risk. Additionally, the independent predictive performance of cardiorespiratory fitness, waist-to-height ratio, and body mass index also were estimated and compared.

**Methods:**

This was a prospective cohort study of 10,381 men who had a normal electrocardiogram and no history of major chronic disease at baseline from 1979 to 2005. Random survival forest models and traditional Cox proportional hazards models were used to predict diabetes at 5-, 10-, and 15-year incidence horizons.

**Results:**

Overall, 4.8% of the participants developed diabetes. Receiver operating characteristic curve analyses for incidence risk demonstrated good discrimination using random survival forest models across fitness and fatness measures; Cox models were poor to fair. The differences between fitness and fatness measures across horizons were clinically negligible. Smoothed random survival forest estimates demonstrated the impact of each fitness and fatness measure on incident diabetes was intuitive and graded.

**Conclusions:**

Although fitness and fatness measures showed a similar discriminative ability in predicting incident diabetes, unique to the study was the ability of the fit-fat index to demonstrate a better indication of incident risk when compared to fitness or fatness alone. A single index combining cardiorespiratory fitness and waist-to-height ratio may be more useful because it can indicate improvements in either or both of the measures.

## Introduction

According to the World Health Organization, the prevalence of diabetes has increased fourfold in the last 35 years affecting 422 million people with an estimated annual total cost of US $827 billion[1]. Along with a better understanding of the gaps in research, the World Health Organization has called for prevention efforts focusing on diet, physical activity, and weight. To help support prevention efforts, a more upstream and nuanced understanding of the influence of common health-related fitness measures is needed to help individuals assess their future diabetes risk.

Measures of fitness and fatness have been found to be associated with diabetes [2–4]. Two fitness and fatness measures linked to diabetes risk are cardiorespiratory fitness (CRF) [4] and waist-to-height ratio (WHtR) [5]. Cardiorespiratory fitness represents the maximum capacity of the cardiorespiratory system to take up and use oxygen, and it is expressed in maximal metabolic equivalents (METs) [6]. Cardiorespiratory fitness is a trait measure modifiable by changes in physical activity, smoking, and body weight [7]. Waist-to-height ratio is an anthropometric indicator of visceral fatness linked to lifestyle factors such as sleep, stress, sedentary behavior, physical activity, muscular fitness, energy balance, and diet quality [8–10]. A key advantage of WHtR when compared to other anthropometric measures is its universal risk threshold; therefore, it is not limited to classification issues of age, gender, ethnicity, or phenotype [11]. Recent systematic reviews and meta-analyses have provided evidence for WHtR as a good predictor of diabetes risk [5, 12].

The scope of evidence indicates an interplay between fitness and fatness regarding incident diabetes risk. It is unclear which plays a more important role or whether they are equally important [2–4, 13]. To provide more clarity, clinicians and researchers have called for the use of robust and objective measures, focusing more on the joint contributions of fitness and fatness in healthy and at-risk individuals [2–4, 13]. Compared to independent analyses, joint categorical analyses can reveal an interplay between fitness and fatness because in joint categorical analyses, individuals are classified as fit/fat, fit/lean, unfit/lean, and unfit/fat [3]. Joint investigations have shown that individuals in the unfit/fat category carry the highest risk for diabetes, while individuals in the fit/fat category carry an equivalent or higher risk for incident diabetes compared to individuals in the unfit/lean category [2, 3, 6, 14, 15]. Joint categorization is somewhat limited because it does not allow for the detection of modest intra-individual changes and may reveal only large-scale shifts in risk. Our study provides a more in-depth examination of a fitness and fatness joint analysis previously conducted in men [6].

To date, there are no studies on the feasibility of an index that can measure the impact of combined relative levels of fitness and fatness on diabetes risk. A single index combining fitness and fatness could be very useful in practice because it can indicate improvements in one or both of the measures. Such an index may assist in helping individuals better understand their risk, set personalized goals, and sustain motivation.

The primary aim of this study was to investigate the utility of a fit-fat index (FFI) by combining CRF and WHtR for the prediction of incident diabetes risk. Secondarily, we aimed to provide clarity on the contributions of conventional measures of fitness and fatness to incident diabetes risk comparing machine learning versus a standard practice regression method.

## Methods

### Study Population

The main analysis was based on apparently healthy men at baseline in the Aerobics Center Longitudinal Study (ACLS). The ACLS cohort included 10,381 individuals, primarily Caucasian, college-educated men, ranging from 20 to 100 years of age, who participated in at least two preventive health examinations at the Cooper Clinic in Dallas, Texas from 1979 to 2005. Detailed information about the cohort has been published elsewhere [16].

### Database

Among the 12,834 men with at least two visits, 2,085 with abnormal electrocardiograms; histories of myocardial infarctions, strokes, or cancers; body mass indexes of (BMI) <18.5 kg/m^2^; or failure to achieve at least 85% of maximal heart rate (220 minus age in years) during a graded exercise test were excluded. Men with physician-diagnosed diabetes, fasting plasma glucose 7.0 mmol/L, or a history of insulin therapy were also excluded (*n* = 368). All participants provided written informed consent for the clinical examinations. This study has been reviewed and approved annually by the Institutional Review Board at the Cooper Institute.

### Measurements

Ascertainment of diabetes occurred during follow-up examinations, which, according to American Diabetic Association criteria, is defined as fasting plasma glucose concentration ≥ 7.0 mmol/L, physician diagnosis of diabetes, or history of insulin therapy. Each participant was tracked from the baseline examination to the first follow-up examination where the individual was identified with diabetes or the last follow-up observation without diabetes. Each preventive health examination was completed after a 12-hour fast. Details of the full examinations have been described previously [16]. A standardized medical questionnaire was used to ascertain demographic information, lifestyle habits, and chronic-disease status. Resting blood pressure was measured by standard auscultatory methods after at least 5 minutes of seated rest and recorded as the average of two or more readings separated by 2 minutes. Cardiorespiratory fitness was expressed as estimated METs based on total duration of a symptom-limited maximal modified Balke graded exercise test. The exercise test is highly correlated with maximal cardiopulmonary exercise tests in men (*r* = 0.92) [17]. Detailed information on the graded exercise test is available in prior publications [18]. The BMI was calculated from measured height and weight as kg/m^2^, and waist circumference was measured at the level of the umbilicus with a nonelastic tape measure. Waist-to-height ratio was calculated by dividing the waist in cm by height in cm. The FFI was calculated by dividing METs by WHtR. Scores commonly range from 10–50 on a continuous scale, with higher scores being better.

### Statistical Analyses

Predictive performance of candidate models was assessed and compared at incidence horizons of 5, 10, and 15 years in terms of (a) ability to *discriminate* between persons who would develop diabetes within the incidence horizon and those who would not via the area under the receiver operating characteristic curve (AUC), and (b) *calibration* of predicted probability of developing diabetes to observed incidences via square root Brier scores. Larger AUCs indicate a more discriminating model, while smaller Brier scores indicate a better-calibrated model. Time-specific AUCs and Brier scores were estimated in the presence of right-censored time to diabetes using the techniques of Uno et al. and Gerds et al., respectively[19, 20]. Predictive performance of FFI, WHtR, BMI, and CRF were estimated both individually and in the context of models adjusted by age and examination year. Upper benchmark models, representing near-best estimates of the relationships of all available covariates (those summarized in Table 1) with incident diabetes and all available non-blood-based covariates (those marked with ^#^ in Table 1) with incident diabetes, were generated to provide reference standards. Two types of prediction models were entertained: (a) Cox proportional hazards models with stepwise Bayesian information criterion variable selection starting from a null model having no included variables and (b) random survival forest models. The random survival forest is a machine learning prediction tool adapted to right-censored time-to-event data. In brief, predictions are based on *bagged*, or bootstrap aggregated, survival predictions over a large number of regression trees. Each regression tree is formed via a *recursive partitioning* of the covariate space, where each split represents the maximum log-rank statistics across split points and among $\sqrt{total\;number\;of\;covariates}$ randomly selected covariates [21]. Separately, multivariate analyses were conducted to demonstrate traditional hazard ratios (S2 Table).

**Table 1:** Baseline characteristics of the aerobics center longitudinal study cohort 1979–2005.

Characteristic		All participants *n* = 10,381	Men *with* Diabetes* *n* = 216	Men *without* diabetes* *n* = 5,191	Men censored* *n* = 4,974
Age (years)^#^		43.3 (8.9)	47.4 (8.5)	42.5 (8.8)	44.1 (8.9)
Glucose (mmol,L)		5.5 (0.5)	5.8 (0.6)	5.4 (0.5)	5.5 (0.5)
Total cholesterol (mmol/L)		11.5(2.2)	11.6 (2.2)	11.5 (2.2)	11.5 (2.2)
HDL-cholesterol (mmol/L)		2.6 (0.7)^a^	2.4 (0.6)^b^	2.6 (0.7)^c^	2.6 (0.7)^d^
Resting systolic blood pressure (mmHg)^#^		120.0 (12.4)	123.7 (13.1)	119.0 (12.1)	120.9 (12.5)
Resting diastolic blood pressure (mmHg)^#^		80.7 (9.3)	83.1 (10.3)	79.9 (9.0)	81.4 (9.4)
Baseline examination year^#^		1989.2 (7.3)	1992.9 (7.7)	1987.6 (6.1)	1990.7 (7.9)
Current smoker^#^		1,551 (14.9%)	36 (16.7%)	688 (13.3%)	827 (16.6%)
Current heavy drinker^#^^,^^e^		1,766 (17.0%)	35 (16.2%)	899 (17.3%)	832 (16.7%)
IFG^f^ at baseline (mmol/L)		4,351 (41.9%)	143 (66.2%)	2,116 (40.8%)	2,092(42.1%)
Family history of diabetes^#^		806 (7.8%)	29 (13.4%)	353 (6.8%)	424 (8.5%)
Waist (cm)^#^		93.0 (9.5)	96.8 (10.3)	92.2 (9.2)	93.7 (9.8)
Height (cm)^#^		179.4 (6.5)	179.7 (7.0)	179.4 (6.5)	179.4 (6.6)
Weight (kg)^#^		84.5 (12.3)	89.1 (13.3)	83.3 (11.7)	85.5 (12.8)
BMI (kg/m^2^)^#^		26.2 (3.3)	27.6 (3.7)	25.9 (3.1)	26.5 (3.5)
CRF (METs)^#^		12.3 (2.3)	11.3 (2.2)	12.6 (2.3)	12.0 (2.2)
WHtR (Waist/Height cm)^#^		0.52 (0.05)	0.54 (0.06)	0.51 (0.05)	0.52 (0.05)
FFI (METs/WHtR)^#^		24.1 (6.2)	21.5 (5.8)	24.9 (6.3)	23.4 (6.1)
CRF category^#^^,^^g^	1 (Low)	879 (8.5%)	28 (13.0%)	364 (7.0%)	487 (9.8%)
	2	1,790 (17.2%)	42 (19.4%)	811 (15.6%)	937 (18.8%)
	3	2,117 (20.4%)	54 (25.0%)	1,022 (19.7%)	1,041(20.9%)
	4	2,764 (26.6%)	51 (23.6%)	1,379 (26.6%)	1,334(26.8%)
	5 (High)	2,831 (27.3%)	41 (19.0%)	1,615 (31.1%)	1,175(23.6%)
Leisure time physical activity category^#^^,^^h^	Inactive	6,313 (60.8%)	107 (49.5%)	3,314 (63.8%)	2,892(58.1%)
	Somewhat active	1,512 (14.6%)	41 (19.0%)	711 (13.7%)	760 (15.3%)
	Active	2,556 (24.6%)	68 (31.5%)	1166 (22.5%)	1,322(26.6%)

*Diabetes/censoring status at median follow-up (4.1 years).

^#^Non-blood-based covariates.

Quantitative variables are summarized as mean (SD); categorical variables are summarized as number (percent). IFG = insulin fating glucose. BMI = body mass index. CRF = cardiorespiratory fitness. METs = metabolic equivalents. WHtR = waist-to-height ratio. FFI = fit-fat index

^a^50,

^b^1,

^c^44, and

^d^5 missing values;

^e^ >14 alcoholic drinks per week;

^f^insulin Fasting Glucose 5.6–6.9 mmol/L;

^g^categories based on quintiles (low fitness: quintile 1; moderate fitness: quintile 2 and 3; high fitness: quintile 4 and 5) of maximal treadmill time in age group (20–39, 40–49, 50–59, 60+) among the overall ACLS population;

^h^Inactive: 0 MET-min per week, Somewhat active: 1–499 MET-min per week, Active: ≥ 500 MET-min per week.

All analyses were based on estimates pooled across three complete datasets formed via predictive mean matching multiple imputation. In brief, each multiply imputed dataset was formed by looping through the variables several times. At each step of the loop, the non-missing values of the current variable of interest were regressed (with an outcome appropriate model) on the remaining variables, a *noisy* prediction was generated for each of the missing values as a function of the remaining variables, and the *noisy* prediction was replaced with the nearest non-missing value of the variable of interest.

Interval estimates were generated and variances of point estimates reduced by averaging across 20 bootstrap samples, nested within each of the three multiply imputed complete datasets. Generalizability of predictive models’ performance measures, AUC, and square root Brier score were assessed via 10 random iterations of five-fold cross-validation nested within bootstrap samples *within* multiply imputed datasets. To show the impact of both age and fitness or fatness measure for an examination year balancing recency and stability, estimates of incidence for the examination year 2000 were constructed by smoothing random forest estimates for persons aged 25, 45, and 65 years. Point wise, 95% confidence intervals were generated by smoothing the relevant quantiles over bootstrap samples.

All analyses were performed in R 3.1.1 (R Foundation for Statistical Computing, Vienna, Austria). Multiple imputation was performed using the package mi, [22] random survival forest models were fit using the package randomForestSRC, [21] time-specific AUCs were estimated using the package survAUC, [23] and several computationally expensive aspects of the analysis were parallelized using functionality in the R packages foreach [24] and doParallel [25].

## Results

Median follow-up was 4.1 years (range 0.1–26.3 years); 4.8% of the participants developed incident diabetes eventually, and 7.8% of the participants had a family history of diabetes. Participant baseline characteristics overall and according to diabetes or right censoring status at median follow-up are shown in Table 1. Missing measurements in the analysis data were limited to 50/10,381 (0.5%) missing HDL values.

Broadly, discriminative ability in terms of AUC and calibration for 15-year incidence horizon was substantially better for random survival forest models than for Cox proportional hazards models with Bayesian information criterion variable selection. Results for 5 and 10 years were similar (S1 Table). Based on all variables, the upper benchmark random survival forest model had cross-validated AUC of 90.4 (95% CI 85.1–93.6). Age and examination year adjusted AUCs ranged from 86.2 (95% CI 82.6–88.7) for CRF to 87.4 (95% CI 82.2–90.4) for FFI (Table 2). The differences between fitness and fatness measures across incidence horizons appeared to have little clinical meaningfulness (S1 Table). Random survival forest models were well calibrated with 15-year incidence horizon square root Brier scores (×100) for age, examination year, and each fitness and fatness measure ranging from 5.6 (95% CI 5.2–6.1) for WHtR to 6.2 (95%, CI 5.6–7.1) for CRF. Results for 5 and 10 years were similar (S1 Table). Traditional hazard ratios indicated FFI to be associated with the lowest risk of diabetes across fitness fatness measures, 0.53 (0.39–0.72) (S2 Table).

**Table 2:** Cross-validated AUC (×100, 95% confidence interval) for the random survival forest model based on the complete aerobics center longitudinal study cohort at 15-year incidence horizon by fitness and fatness characteristics.

	Unadjusted AUC	Age- and exam-year-adjusted AUC
FFI	80.5 (70.5–83.7)	87.4 (82.2–90.4)
WHtR	78.7 (71.0–84.4)	87.3 (82.1–90.6)
BMI	76.6 (72.0–83.0)	86.6 (82.3–88.8)
CRF	55.3 (48.7–62.6)	86.2 (82.6–88.7)

AUC = area under the receiver operating characteristic curve. FFI = fit-fat index. WHtR = waist-to-height ratio. BMI = body mass index. CRF = cardiorespiratory fitness. Column 1 provides AUCs for individual fitness and fatness measures and Column 2 provides AUCs for fitness and fatness measures in models adjusted for age and examination year.

**Fig 1:**
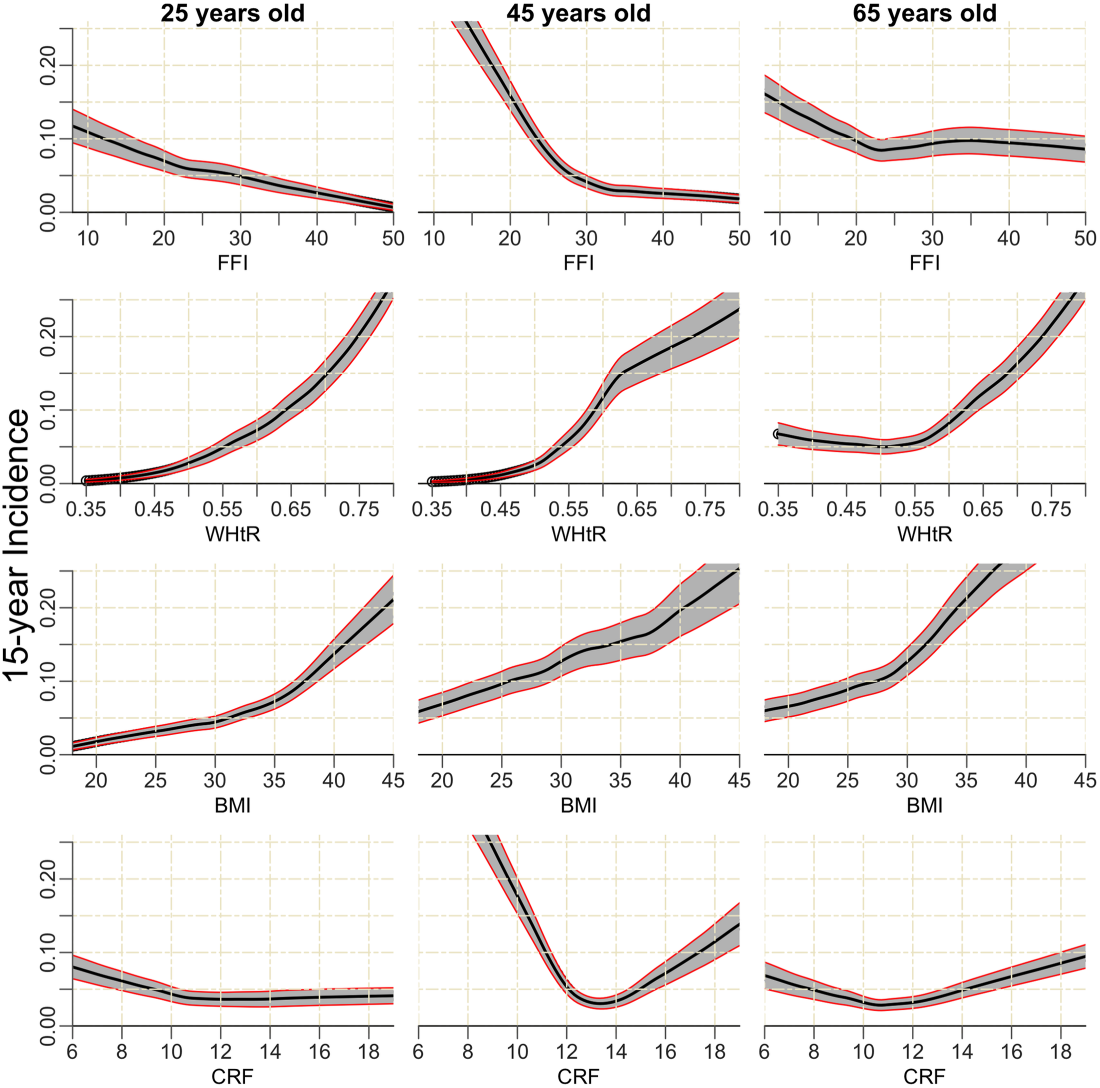
Smoothed random survival forest predictions for 15-year diabetes incidence vs. fitness and fatness measures. 15-year incidence of diabetes in black, 95% pointwise confidence intervals in red/grey. Predictions are averaged over men aged 25-, 45-, and 65-years-old with examination year 2000 and smoothed.

Smoothed random survival forest estimates of 15-year incidence for men in the examination year 2000, aged 25, 45, and 65 years, are shown in Fig 1. Broadly, the impact of fitness and fatness on incident diabetes was intuitive and graded. For example, men in exam year 2000, ages 25 or 45, with an FFI >30 had a 15-year incidence of diabetes <5%, while 25-year-old men with FFI <12, 45-year-old men with FFI <23, and 65-year-old men with FFI <18 had a 15-year incidence of diabetes >10%. Results for 5 and 10 years were similar (S1 Fig).

## Discussion

Overall, the results indicated objectively measured fitness and fatness are important predictors of incident diabetes. The age- and examination-year-adjusted models achieved an ideal balance of parsimony and high accuracy and were capable of representing the complex interactions among age, examination year, and fitness or fatness measure. The upward hinge in diabetes risk across CRF was unexpected for the 45-year-old men, but this risk across CRF appears to be genuine (S1 File). To date, only one previous study used a receiver operating characteristic analysis to compare fitness and fatness with 15.5-year incident diabetes [26] in a modest sample -size group (*n* = 1543) of men and women. The results showed muscular fitness (AUC = 0.74), but not CRF (AUC = 0.63), had similar discriminative ability to BMI (AUC = 0.71) and waist circumference (AUC = 0.72) for predicting incident diabetes. Reports and researchers have suggested fitness and fatness [3, 6, 26] are equal contributors, but findings may have been limited because inference was based on a comparison of hazard ratios in the context of Cox models. In a recent study of Japanese men (*n* = 3523), researchers suggested BMI has a greater role than CRF, based on joint associations for incident diabetes via hazard ratios in a Cox model [14]. Our study indicates a traditional Cox proportional hazards model, combined with stepwise variable selection, could not capture the complex relationship between fitness and fatness measures and diabetes incidence, particularly in the presence of important adjustment variables such as age and year of examination. Additionally, the Japanese findings may have been biased because of a less-accurate measure of CRF and, likely, a misclassification of fatness using BMI rather than WHtR for Japanese men [27].

Although FFI and other fitness fatness measures showed similar discriminative ability in predicting incident diabetes, the advantage of FFI is apparent when considering incident risk. Unique to the study was the ability of FFI to show lower (higher) incident risk in cases in which independent measures of fitness or fatness imply a higher (lower) risk. The 15-year incidence estimates from Fig 1, which are shown in Table 3, provides an illustration of the potential value of the FFI for a 45-year-old man. For instance, if only being less fat was considered, his risk would be low (2%), but if he were less fit/less fat, his risk would be substantially higher (10%). On the other hand, if he were less fit/more fat, his diabetes risk would be the highest (20%). Table 3 also shows the importance of considering fitness and fatness on a continuum by demonstrating individuals can have differing combined levels of fitness and fatness but can have equivalent FFI (≈23) incident diabetes risk (10%). Overall, the example shows fitness or fatness alone may be less useful without consideration of their interplay. From another perspective, FFI provides two measures by which risk of diabetes may be modified. To our knowledge, this is the first study to evaluate the combined association of CRF and WHtR expressed in a unique index with incident diabetes in a large cohort of apparently healthy men at baseline.

**Table 3:** Approximate 15-year incidence random survival forest estimates for examination year 2000, 45-year-old man by fitness and fatness measures (see Fig 1).

		**WHtR		
		0.48 Less Fat	0.60 More Fat	
*METs	11 Less Fit	10% (FFI≈23)	20% (FFI≈18)	11%*
	14 More Fit	5% (FFI≈29)	10% (FFI≈23)	4%*
		2%**	12%**	

WHtR = waist-to-height ratio. METs = metabolic equivalents. FFI = fit-fat index.

*Represents independent risk for MET level.

**Represents independent risk for WHtR level.

Although no researchers have investigated the relationship of combined CRF and WHtR in the form of FFI, researchers have documented the categorical joint association of CRF and fatness with incident diabetes [6, 14]. Lee and colleagues found men in the fit/fat (waist>102 cm) category had a hazard ratio of 2.38 (1.78–3.17) for diabetes, whereas the unfit/lean had a lower hazard ratio of 1.77 (1.07–2.93) when compared with the fit/lean category. A similar relationship was seen in Korean adults, using a ratio of visceral to subcutaneous fat ≥ 4 to define fatness. Compared to the fit/lean category, the unfit/lean, and fit/fat categories had comparable risk of metabolic syndrome [15]. Last, in an older adult diabetic randomized-control trial (mean age 57 years, 37% men), interplay between fitness and fatness was indicated. Senechal and colleagues found those with any increase in CRF (change in estimated METs >0) and any decrease in waist circumference (change in cm <0) had higher odds 2.81 (1.13–6.81) of achieving a clinically relevant decrease (0.5%) in HbA1c [28]. The authors summarized a program for individuals with diabetes should emphasize increasing fitness and decreasing central obesity. Collectively, our findings, along with the related evidence, indicate improvements in fitness or fatness may reduce the risk of diabetes, and the use of FFI may be more advantageous.

Biological mechanisms that improve fitness, which may reduce the risk of incident diabetes, include structural (increases in muscle fiber size, percent of type IIa fibers, and capillary density) and biochemical changes (improvements in insulin-signaling kinetics, enzyme action, and myoglobin) in skeletal muscles to improve insulin sensitivity and glucose homeostasis. Decreased levels of central obesity may affect insulin metabolism by decreasing the release of free fatty acids. Excess free fatty acids reduce the hepatic clearance of insulin, which may lead to insulin resistance, hyperinsulinemia, and diabetes. It is important to note fitness and central obesity are interrelated to some extent because improvements in aerobic and/or muscular fitness may reduce central fatness without changes in BMI [8, 29]. The strengths of our study include validated and objective measurements of CRF, waist, height, and weight. Furthermore, incident diabetes was measured objectively at baseline and follow-up examinations, likely resulting in a low rate of misclassification. Limitations of the present study included a lack of information about the type of diabetes; therefore, Type 1 (insulin-dependent) and Type 2 diabetes could not be differentiated. Insulin use, however, was recorded on the last follow-up medical questionnaire among men who were diagnosed with diabetes on the basis of fasting plasma, and only 2.7% (13 of 477) reported insulin use. There are likely few men with Type 1 diabetes present in this study because none were diagnosed with diabetes at age <30 years.

The generalizability of the findings is limited because participants were mostly well-educated Caucasian men. In future studies, we plan to test the relevancy of FFI for diabetes risk in women and different ethnic groups. On the other hand, the homogeneity of the sample strengthened the internal validity of our findings by limiting possible socioeconomic confounders. The biological processes underlying the roles of CRF and WHtR in the etiology of diabetes are likely similar across socioeconomic and ethnic groups [29]. Notably, although the prevalence of diabetes in the United States is higher in non-Caucasians, officials at the Centers for Disease Control and Prevention, National Center for Health Statistics reported Caucasian males had the greatest age-adjusted prevalence increase (from 2.5% to 6.5%) from 1980 to 2011 [30]. Although data from an oral glucose tolerance test were not available, a fasting plasma glucose test is an objective and validated basis for diabetes diagnosis, as endorsed by the American Diabetes Association and the World Health Organization, and is used in numerous prospective studies. Additionally, participants underwent regular follow-ups (on average, every 1.5 years), and fasting glucose tests were likely to identify most diabetes events. Diet-quality and energy-intake data were not available.

## Conclusions

In summary, our findings add to and expand upon the limited body of evidence regarding the relationship of fitness and fatness with incident diabetes. We found both fitness and fatness have a comparable discriminative ability in predicting diabetes risk in a large cohort of apparently healthy Caucasian men. Unique to the study was the ability of FFI to provide a more comprehensive indication of incident diabetes when compared to fitness or fatness alone. A single index may be more useful because it can account for the degree of change in CRF and/or WHtR. Future studies should examine FFI utility among women and different race-ethnicities, as well as the practical adaptation of the index and its impact on attaining and maintaining healthy lifestyle practices.

## Supporting Information

S1 Fig 5 and 10-year incident predictions. (DOCX)

S1 File CRF hinge. (DOCX)

S1 Table 5 and 10-year Cross-validated AUC’s and square root Brier scores. (DOCX)

S2 Table Traditional Cox model. (DOCX)
