# Supplementary material for: A Fit-Fat Index for Predicting Incident Diabetes in Apparently Healthy Men: A Prospective Cohort Study
Source: PLoS One. 2016 Jun 24;11(6):e0157703. doi: 10.1371/journal.pone.0157703 (PMC4920380; doi:10.1371/journal.pone.0157703)
Supplement: S1 Fig — (DOCX) [file pone.0157703.s001.docx]

Supplementary Figures 1


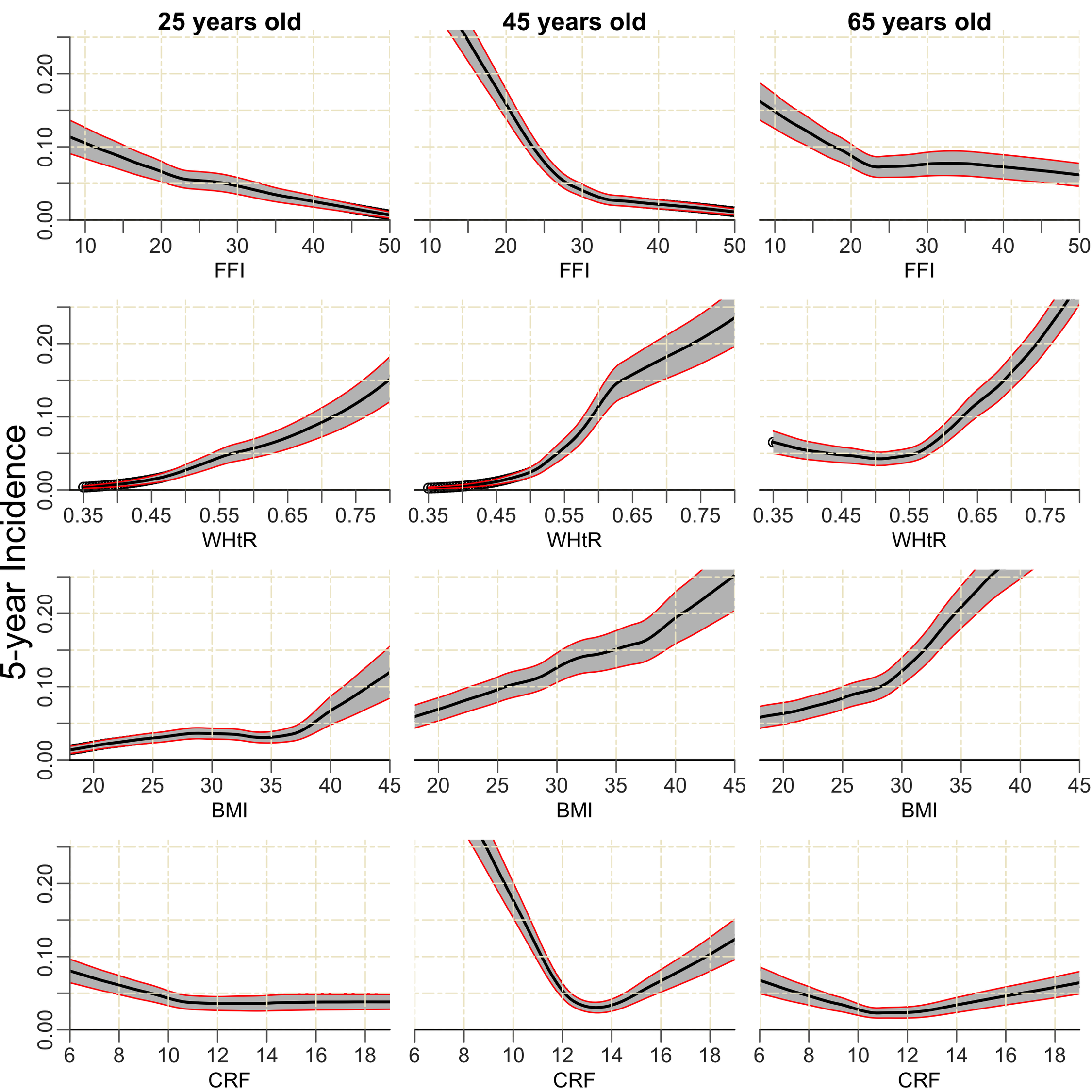


Smoothed predictions for 5-year diabetes incidence vs. fitness and fatness measures. Predictions are averaged over men aged 25, 45 and 65 years old with exam year 2000 and smoothed. 5-year incidence of diabetes in black, 95% pointwise confidence intervals in red/grey.


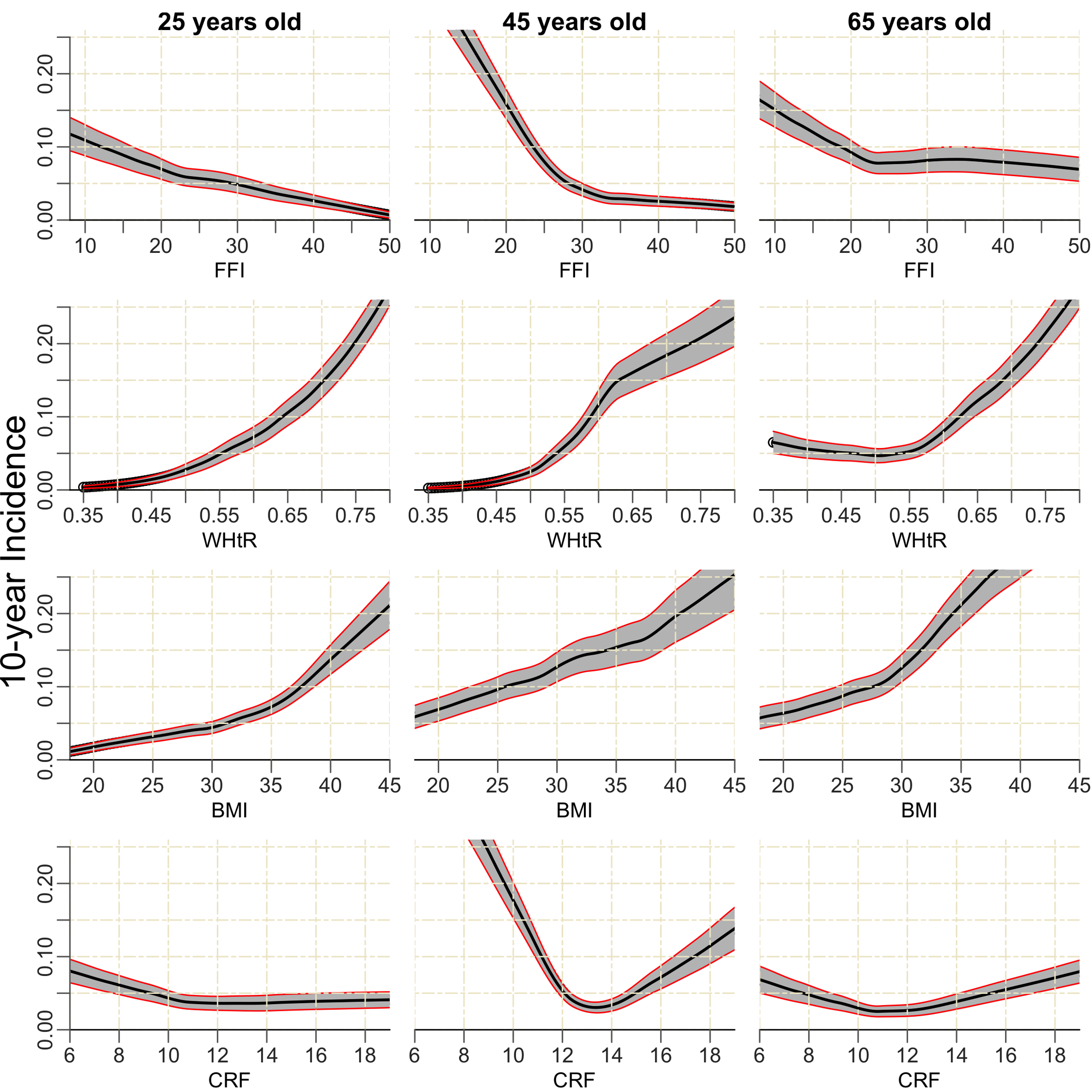


Smoothed predictions for 10-year diabetes incidence vs. fitness and fatness measures. Predictions are averaged over men aged 25, 45 and 65 years old with exam year 2000 and smoothed. 10-year incidence of diabetes in black, 95% pointwise confidence intervals in red/grey.
