# Supplementary material for: A Fit-Fat Index for Predicting Incident Diabetes in Apparently Healthy Men: A Prospective Cohort Study
Source: PLoS One. 2016 Jun 24;11(6):e0157703. doi: 10.1371/journal.pone.0157703 (PMC4920380; doi:10.1371/journal.pone.0157703)
Supplement: S1 File — (DOCX) [file pone.0157703.s002.docx]

S1 File

The unexpected relationship between CRF and diabetes incidence in 45-year-old men was examined from two additional perspectives. First, Kaplan-Meier incidence estimates were generated for men ages 40–50 years old within exam years 1995–2005 by CRF category. For CRF ≤10, the 5-year incidence was estimated at approximately 12%, for 10< CRF ≤12 was at 7%, for 12< CRF ≤15 was at 4%, and for 15< CRF was at 11%. Then, a Cox model restricted to men ages 40–50 years old within exam years 1995–2005 was fitted to CRF category. With CRF ≤ 10 as the reference group, estimated hazard ratios were 0.56 (p=0.07), 0.30 (p<0.01), and 0.91 (p=0.83), for 10< CRF ≤12, 12 < CRF ≤15, and 15< CRF groups, respectively. Essentially, both analyses were consistent with the random forest estimates. From another perspective, the generalizable accuracy of the random forest model was assessed using cross-validation. The random forest model was shown to have high accuracy on independent testing data (i.e., data that were not used in the fitting process). Evidence exists that a complex interaction between CRF, age, and exam years was present. Further examination of the trend across populations is warranted.
