# Supplementary material for: A Fit-Fat Index for Predicting Incident Diabetes in Apparently Healthy Men: A Prospective Cohort Study
Source: PLoS One. 2016 Jun 24;11(6):e0157703. doi: 10.1371/journal.pone.0157703 (PMC4920380; doi:10.1371/journal.pone.0157703)
Supplement: S1 Table — (DOCX) [file pone.0157703.s003.docx]

**Supplementary Tables 1**

Cross-validated AUCs (×100, 95% confidence interval) for the upper benchmark, nonblood variables, fitness and fatness variables without
adjustment and adjusted for age and exam year.

| Model | Cox proportional hazards model | | | Random survival forest model | | |
| --- | --- | --- | --- | --- | --- | --- |
| Incidence Horizon | 5 year | 10 year | 15 year | 5 year | 10 year | 15 year |
| Upper Benchmark | 84.9 (80.4–87.9) | 80.6 (75.4–84.7) | 74.0 (68.8–79.0) | 93.6 (89.2–95.5) | 91.2 (86.9–93.4) | 90.4 (85.1–93.6) |
| Non–Blood Variables | 78.3 (74.5–82.1) | 80.0 (74.3–84.2) | 74.0 (69.1–79.4) | 89.3 (85.9–92.7) | 88.7 (83.1–93.1) | 90.5 (84.2–94.1) |
| FFI† | 79.8 (75.2–84.2) | 78.9 (74.5–82.0) | 73.9 (70.7–77.8) | 86.2 (81.1–89.8) | 84.8 (81.5–90.0) | 87.4 (82.2–90.4) |
| WHtR† | 79.3 (75.1–83.6) | 79.6 (75.8–82.9) | 77.6 (73.2–80.8) | 85.7 (78.6–90.2) | 86.4 (83.9–91.3) | 87.3 (82.1–90.6) |
| BMI† | 79.2 (75.2–83.7) | 79.4 (74.2–83.0) | 76.8 (73.2–81.8) | 84.7 (80.0–89.4) | 85.8 (82.4–89.8) | 86.6 (82.3–88.8) |
| CRF† | 78.6 (74.3–83.6) | 78.3 (72.9–81.3) | 73.4 (70.1–76.5) | 83.4 (77.0–88.2) | 83.1 (79.3–89.3) | 86.2 (82.6–88.7) |
| FFI | 67.8 (61.2–70.9) | 65.5 (60.7–69.9) | 60.0 (52.9–65.3) | 78.1 (73.4–83.0) | 79.2 (73.5–84.9) | 80.5 (70.5–83.7) |
| WHtR | 64.8 (59.2–69.5) | 66.7 (58.4–73.2) | 64.3 (58.2–69.3) | 78.7 (72.6–84.2) | 78.6 (74.3–84.9) | 78.7 (71.0–84.4) |
| BMI | 65.6 (59.3–70.8) | 65.7 (61.1–71.9) | 65.0 (60.0–70.0) | 78.6 (69.9–83.6) | 76.5 (73.3–81.6) | 76.6 (72.0–83.0) |
| CRF | 64.5 (59.1–70.2) | 63.7 (55.4–71.5) | 58.9 (50.0–66.5) | 66.2 (59.7–72.2) | 63.0 (57.3–69.1) | 55.3 (48.7–62.6) |

AUC=area under the receiver operating characteristic curve. FFI=fit–fat index. WHtR= waist–to–height ratio. BMI=body mass index. CRF=cardiorespiratory fitness. †Adjusted for age, examination year.

Cross-validated square root Brier scores (×100, 95% confidence interval) for the upper benchmark, nonblood variables, fitness and fatness variables without adjustment and adjusted for age and exam year.

| Model | Cox proportional hazards model | | | Random survival forest model | | |
| --- | --- | --- | --- | --- | --- | --- |
| Incidence Horizon | 5 year | 10 year | 15 year | 5 year | 10 year | 15 year |
| Upper benchmark | 3.0 (2.6–3.4) | 5.3 (4.7–5.8) | 8.2 (7.2–9.2) | 1.7 (1.5–1.9) | 3.1 (2.7–3.4) | 5.1 (4.5–5.7) |
| Non–Blood variables | 3.1 (2.7–3.6) | 5.6 (5.1–6.6) | 8.2 (7.1–9.3) | 1.9 (1.6–2.0) | 3.4 (3.0–3.7) | 5.3 (4.6–6.1) |
| FFI† | 3.1 (2.7–3.4) | 5.7 (5.2–6.3) | 8.3 (7.6–9.7) | 1.9 (1.8–2.1) | 3.7 (3.3–4.0) | 5.7 (5.2–6.1) |
| WHtR† | 3.1 (2.8–3.6) | 5.8 (5.1–6.3) | 8.5 (7.8–9.3) | 2.0 (1.7–2.1) | 3.6 (3.4–3.9) | 5.6 (5.2–6.1) |
| BMI† | 3.0 (2.7–3.5) | 5.7 (5.2–6.3) | 8.4 (7.6–9.2) | 1.9 (1.8–2.2) | 3.8 (3.4–4.2) | 5.7 (5.1–6.5) |
| CRF† | 3.0 (2.8–3.3) | 5.7 (5.0–6.3) | 8.4 (7.2–9.6) | 2.2 (2.0–2.4) | 4.3 (3.7–4.6) | 6.2 (5.6–7.1) |
| FFI | 3.2 (2.7–3.6) | 6.4 (5.8–6.9) | 10.1 (8.9–11.2) | 2.5 (2.3–2.8) | 4.7 (4.5–5.3) | 7.2 (6.4–7.9) |
| WHtR | 3.3 (2.8–3.8) | 6.5 (5.8–7.3) | 10.1 (9.3–10.6) | 2.9 (2.6–3.4) | 5.5 (4.9–6.1) | 8.3 (7.5–9.0) |
| BMI | 3.4 (2.8–3.7) | 6.4 (5.9–7.1) | 10.0 (9.0–11.0) | 3.3 (2.9–3.6) | 6.0 (5.6–6.5) | 9.0 (8.4–10.2) |
| CRF | 3.4 (3.1–3.8) | 6.6 (5.8–7.1) | 9.7 (9.3–11.1) | 3.4 (3.1–3.8) | 6.6 (5.8–7.1) | 9.8 (9.2–11.1) |

FFI=fit–fat index. WHtR= waist–to–height ratio. BMI=body mass index. CRF=cardiorespiratory fitness. †Adjusted for age, examination year

Cross-validated p-values for AUC comparisons of variables BMI, FFI, WHtR and CRF (models adjusted for age and exam year).

| Model | Cox proportional hazards model | | | Random survival forest model | | |
| --- | --- | --- | --- | --- | --- | --- |
| Incidence Horizon | 5 year | 10 year | 15 year | 5 year | 10 year | 15 year |
| FFI/ WHtR | 0.015 | <0.001 | <0.001 | 0.175 | <0.001 | 0.229 |
| FFI/BMI | 0.250 | <0.001 | <0.001 | 0.058 | 0.002 | 0.055 |
| FFI/ CRF | <0.001 | <0.001 | 0.004 | <0.001 | <0.001 | 0.017 |
| WHtR/ BMI | 0.131 | 0.532 | 0.109 | 0.949 | 0.282 | <0.001 |
| WHtR/CRF | 0.455 | <0.001 | <0.001 | 0.005 | <0.001 | <0.001 |
| BMI/CRF | 0.250 | <0.001 | <0.001 | <0.001 | <0 .001 | 0.385 |

FFI=fit–fat index. WHtR= waist–to–height ratio. BMI=body mass index. CRF=cardiorespiratory fitness.
