# Supplementary material for: A Fit-Fat Index for Predicting Incident Diabetes in Apparently Healthy Men: A Prospective Cohort Study
Source: PLoS One. 2016 Jun 24;11(6):e0157703. doi: 10.1371/journal.pone.0157703 (PMC4920380; doi:10.1371/journal.pone.0157703)
Supplement: S2 Table — (DOCX) [file pone.0157703.s004.docx]

Table 1. Baseline characteristics of men according to fit-fat index (FFI) levels (quartiles)

| Characteristics | All men | 1st quartile  (Low) | 2nd quartile | 3rd quartile | 4th quartile  (High) | P value ^a^ |
| --- | --- | --- | --- | --- | --- | --- |
| n | 10,381 | 2,593 | 2,598 | 2,594 | 2,596 | - |
| Patients with diabetes (n) | 498 | 179 | 138 | 85 | 96 | - |
| Age (years) | 43.4 ± 8.9 | 47.2 ± 9.2 | 44.5 ± 8.4 | 42.2 ± 8.2 | 39.5 ± 7.9 | <0.001 |
| FFI, median | 23.4 | 17.5 | 21.6 | 25.4 | 31.1 | <0.001 |
| CRF (METs) | 12.3 ± 2.3 | 9.7 ± 1.1 | 11.4 ± 0.8 | 12.8 ± 0.8 | 15.1 ± 1.7 | <0.001 |
| Waist-to-height ratio | 0.52 ± 0.05 | 0.58 ± 0.05 | 0.53 ± 0.03 | 0.50 ± 0.03 | 0.46 ± 0.03 | <0.001 |
| Height (cm) | 179.4 ± 6.5 | 178.3 ± 6.5 | 179.3 ± 6.5 | 179.7 ± 6.5 | 180.2 ± 6.5 | <0.001 |
| Waist circumference (cm) | 93.0 ± 9.5 | 102.5 ± 9.3 | 94.9 ± 6.3 | 90.3 ± 5.6 | 84.4 ± 5.6 | <0.001 |
| Body mass index (kg/m^2^) | 26.2 ± 3.3 | 29.4 ± 3.7 | 26.6 ± 2.4 | 25.3 ± 2.1 | 23.7 ± 1.8 | <0.001 |
| Systolic blood pressure (mmHg) | 120.0 ± 12.4 | 123.1 ± 12.9 | 120.4 ± 12.3 | 118.3 ± 11.8 | 118.3 ± 11.9 | <0.001 |
| Current smokers, n (%) | 1,551 (14.9) | 561 (21.6) | 441 (16.6) | 351 (13.5) | 208 (8.0) | < 0.001 |
| Current heavy drinkers, n (%) | 1,766 (17.0) | 451 (17.4) | 456 (17.6) | 445 (17.2) | 414 (15.9) | 0.404 |

Fit-fat index: CRF (METmax) ÷ waist-to-height ratio; CRF: cardiorespiratory fitness (METs)

Data are means ± SD, unless otherwise specified.

^a^ one-way ANOVA for continuous variables and χ^2^ test for categorical variables.

Table 2. Pearson correlation coefficients among each fitness and fatness variables.

|  | Fit-fat index | Cardiorespiratory fitness | Waist-to-height ratio | Waist circumference |
| --- | --- | --- | --- | --- |
| Cardiorespiratory fitness | 0.95 | ― | ― | ― |
| Waist-to-height ratio | -0.77 | -0.57 | ― | ― |
| Waist circumference | -0.73 | -0.54 | 0.94 | ― |
| Body mass index | -0.64 | -0.47 | 0.88 | 0.86 |

Table 3. Adjusted hazard ratios of incidence of diabetes by potential risk factors

| Potential risk factors | Participants | Hazard ratios | 95%CIs | P value |
| --- | --- | --- | --- | --- |
| Age ^a^  1st quartile (High)  2nd quartile  3rd quartile  4th quartile (Low) | 2,766  2,769  2,332  2,514 | 1.00 (Referent)  0.74  0.72  **0.59** | ―  0.58 - 0.94  0.56 - 0.91  0.45 - 0.76 | ―  0.014  0.007  < 0.001 |
| Fit-fat index ^b^  1st quartile (Low)  2nd quartile  3rd quartile  4th quartile (High) | 2,593  2,598  2,594  2,596 | 1.00 (Referent)  0.83  **0.53**  0.63 | ―  0.65 - 1.06  0.39 - 0.72  0.46 - 0.88 | ―  0.132  < 0.001  0.006 |
| Cardiorespiratory fitness ^c^  1st quartile (Low)  2nd quartile  3rd quartile  4th quartile (High) | 2,496  3,141  1,726  3,018 | 1.00 (Referent)  0.83  0.64  **0.63** | ―  0.66 - 1.05  0.47 - 0.87  0.47 - 0.83 | ―  0.112  0.005  0.001 |
| Waist-to-height ratio ^d^  1st quartile (High)  2nd quartile  3rd quartile  4th quartile (Low) | 2,625  2,625  2,557  2,574 | 1.00 (Referent)  0.70  **0.63**  0.65 | ―  0.55 - 0.89  0.49 - 0.82  0.48 - 0.88 | ―  0.003  < 0.001  0.005 |
| Body mass index ^e^  1st quartile (High)  2nd quartile  3rd quartile  4th quartile (Low) | 2,596  2,596  2,597  2,592 | 1.00 (Referent)  0.71  0.74  **0.62** | ―  0.55 - 0.91  0.56 - 0.97  0.45 - 0.87 | ―  0.006  0.028  0.005 |

a Adjusted for fit-fat index, systolic blood pressure, parental history of diabetes, year of baseline examination, smoking habit, and drinking habit.

b Adjusted for age, body mass index, systolic blood pressure, parental history of diabetes, year of baseline examination, smoking habit, and drinking habit.

c Adjusted for age, body mass index, systolic blood pressure, parental history of diabetes, year of baseline examination, smoking habit, and drinking habit.

d Adjusted for age, cardiorespiratory fitness, systolic blood pressure, parental history of diabetes, year of baseline examination, smoking habit, and drinking habit.

e Adjusted for age, fit-fat index, systolic blood pressure, parental history of diabetes, year of baseline examination, smoking habit, and drinking habit.
